# Ambulante Versorgung des Ulcus cruris venosum in Deutschland: Eine retrospektive Analyse von Abrechnungsdaten

**DOI:** 10.1111/ddg.70021x

**Published:** 2026-10-05

**Authors:** Lisa Weller, Regina Poß‐Doering, Thomas Grobe, Thomas Fleischhauer, Gunter Laux, Jona Frasch, Attila Altiner, Kerstin Protz, Joachim Szecsenyi, Jonas D. Senft

**Affiliations:** ^1^ Qua‐Institut für angewandte Qualitätsförderung und Forschung im Gesundheitswesen GmbH, Göttingen; ^2^ Abteilung Allgemeinmedizin und Versorgungsforschung Universitätsklinikum Heidelberg; ^3^ Universitätsklinikum Hamburg‐Eppendorf, Hamburg; ^4^ Competenzzentrum Versorgungsforschung in der Dermatologie (CVderm) Institut für Versorgungsforschung in der Dermatologie und bei Pflegeberufen (IVDP) Universitätsklinikum Hamburg‐Eppendorf, Hamburg

**Keywords:** Ambulante Versorgung, chronische Wunden, Kompressionstherapie, Ulcus cruris venosum, Chronic wounds, compression therapy, outpatient care, venous leg ulcer

## Abstract

**Hintergrund und Zielsetzung:**

Die ambulante Versorgung von Patienten mit Ulcus cruris venosum (UCV) ist aus medizinischer und gesundheitsökonomischer Perspektive hoch relevant. Häufig erfolgt die Behandlung uneinheitlich und nicht leitliniengerecht. Im Rahmen des GBA‐Innovationsfondsförderprojekts Ulcus Cruris Care (01VSF19043) sollte die ambulante Versorgungslage von Patienten mit UCV in Deutschland untersucht werden.

**Patienten und Methodik:**

Es erfolgte eine retrospektive Sekundärdatenanalyse von 40 046 Patienten mit UCV‐Diagnose anhand von Daten der AOK Baden‐Württemberg der Jahre 2015 bis 2019. Bewertet wurden Verordnungen, Hilfsmittel, Pflege, Nutzung von Kompressionstherapie, operative Eingriffe und Behandlungskosten.

**Ergebnisse:**

47,3 % (n = 7581) der Patienten ohne Kontraindikation erhielten innerhalb von 90 Tagen ein Kompressionshilfsmittel. 2,3 % (n = 135) wurden operativ an Stammvenen behandelt. Nur 14,1 % (n = 771) erhielten eine konsequente Kompressionstherapie, die mit schnellerer Wundheilung (121,6 ± 86,2 vs. 188,1 ± 164,2 Tage, p < 0,001), niedrigeren Gesamtkosten (4280 ± 5326 € vs. 5683 ± 9657 €, p < 0,001) und geringeren krankheitsspezifischen Kosten (2498 ± 2785 € vs. 2797 ± 5145 €, p = 0,017) assoziiert war.

**Schlussfolgerungen:**

Rund 80 % der VLU‐Patienten erhalten keine konsequente Kompressionstherapie und sind somit unzureichend versorgt. Es besteht ein dringender Handlungsbedarf die Behandlungssituation von Menschen mit UCV in Deutschland zu verbessern.

## EINLEITUNG

Die Behandlung chronischer Wunden stellt sowohl medizinisch als auch ökonomisch eine große Herausforderung dar. Bis zu 80 % der chronischen Ulzerationen der unteren Extremität sind auf eine chronisch‐venöse Insuffizienz zurückzuführen.^1^ Die Prävalenz venöser Ulzera in Deutschland wird auf 0,7 %–1,4 % geschätzt und nimmt in Altersgruppen über 65 Jahren deutlich zu.^2^, ^3^ Aufgrund des demografischen Wandels ist künftig mit einer Verschärfung des Versorgungssituation zu rechnen. Neben den physischen, psychischen und sozialen Krankheitsfolgen für die Betroffenen ist das Ulcus cruris venosum (UCV) mit hohen gesundheitsökonomischen Kosten und Arbeitsausfällen verbunden.^2^, ^3^ Laut Versorgungsanalysen entfallen 1 %–2 % des gesamten Gesundheitsbudgets auf die Behandlungskosten.^4^

In Deutschland empfiehlt die kürzlich überarbeitete konsensbasierte Leitlinie zur Diagnostik und Therapie venöser Beinulzera eine frühzeitige und konsequente Kompressionstherapie für Patienten mit UCV, sofern keine Kontraindikationen vorliegen.^1^, ^5^ Sie ist ein zentraler Bestandteil der konservativen Behandlung, deren Wirksamkeit auf die Wundheilung bei fachgerechter Anwendung mit einem Anlagedruck von 40–60 mmHg durch Evidenzklasse IA belegt ist.^6^ Ihre konsequente und sachgerechte Anwendung ist mit einer geringeren Rezidivrate, einer Schmerzreduktion und einer Verbesserung der gesundheitsbezogenen Lebensqualität assoziiert.^6^, ^7^ Dennoch wird die Kompressionstherapie laut Versorgungsanalysen nur in etwa 31 %–61 % der Fälle eingesetzt, wobei auch Defizite in der praktischen Umsetzung bestehen.^8^, ^9^

Das GBA‐Innovationsfondsförderprojekt Ulcus Cruris Care (Nr. 01VSF19043) zielt darauf ab, auf Grundlage aktueller Leitlinien^1^, ^10^, ^11^ und wissenschaftlicher Erkenntnisse ein evidenzbasiertes, patientenorientiertes Versorgungskonzept zur Behandlung des UCV in der hausärztlichen Praxis zu entwickeln und umzusetzen. Im Rahmen des Projekts wurden Routinedaten der gesetzlichen Krankenkasse AOK Baden‐Württemberg ausgewertet, um die aktuelle ambulante Versorgungssituation zu untersuchen. Ziel der vorliegenden Analyse war insbesondere die Erfassung des Einsatzes der Kompressionstherapie als zentrales Behandlungselement, ärztlicher Konsultationen, relevanter operativer Eingriffe sowie der insgesamt entstandenen Kosten. Die Ergebnisse dieser Studie können zur Umsetzung geeigneter Interventionen beitragen und damit die ambulante Versorgung von Patienten mit UCV verbessern.

## PATIENTEN UND METHODIK

### Datengrundlage

Zur Analyse der ambulanten Versorgung wurden Routinedaten der AOK Baden‐Württemberg aus den Jahren 2015 bis 2019 von erwachsenen Versicherten bereitgestellt und analysiert, die ambulant mindestens eine UCV‐Diagnose aufwiesen (ICD‐10‐Codes: I83.0, I83.2, I87.01 oder I87.21). Der verwendete Datensatz umfasste Versichertenstammdaten, ambulante und stationäre Versorgungsdaten, Daten zu Arznei‐ und Verbandmittelverordnungen, Heil‐ und Hilfsmittelverordnungen sowie zu Pflegeleistungen. Die Studie wurde von der Ethikkommission der Medizinischen Fakultät Heidelberg genehmigt (Aktenzeichen S‐489/2021, 24. Juni 2021).

### Identifikation von Krankheitsfällen

Um inzidente Erkrankungsfälle zu identifizieren, wurden Daten zu ambulanten Diagnosen, Verbandmittelverordnungen sowie EBM‐Abrechnungsziffern kombiniert (Abbildung 1). Bei einem UCV finden in der Regel engmaschige Arztkontakte mit regelmäßigen Verordnungen von Verbandmitteln statt. Erkrankungsfälle wurden daher basierend auf den tagesgenau verfügbaren Daten zur Verbandmittelverordnung und Abrechnung der EBM‐Ziffer „02312“ („Behandlungskomplex eines oder mehrerer chronisch venösen/r Ulcus/Ulcera cruris“) bestimmt. Als Startpunkt eines inzidenten Erkrankungsfalls wurde das erste Datum gewählt, zu dem entweder die Ziffer 02312 abgerechnet oder ein Verbandmittel verordnet wurde, sofern im selben Quartal auch eine UCV‐Diagnose im ambulanten Bereich vorlag. Um Verbandmittel zu identifizieren, wurde eine Liste mit mehr als 12 000 der gebräuchlichsten Produkte mit Pharmazentralnummern (PZN) angelegt. Als inzident Erkrankte wurden Betroffene dann identifiziert, wenn nach dem Startdatum mindestens ein weiteres Mal Verbandmittel verordnet wurden beziehungsweise die Ziffer 02312 abgerechnet wurde über einen Zeitraum von mindestens 56 Tagen. Zur Abgrenzung von Erkrankungsepisoden wurde ein Zeitraum von 91 Tagen gewählt, in dem keiner der zwei Kriterien dokumentiert sein durfte. Da die Auswertung auf der (möglichen) Verfügbarkeit ambulanter Daten beruht, wurden Versicherte mit einem langen Krankenhausaufenthalt (> 21 Tage) innerhalb der jeweiligen Beobachtungszeit ausgeschlossen.

**ABBILDUNG 1:**
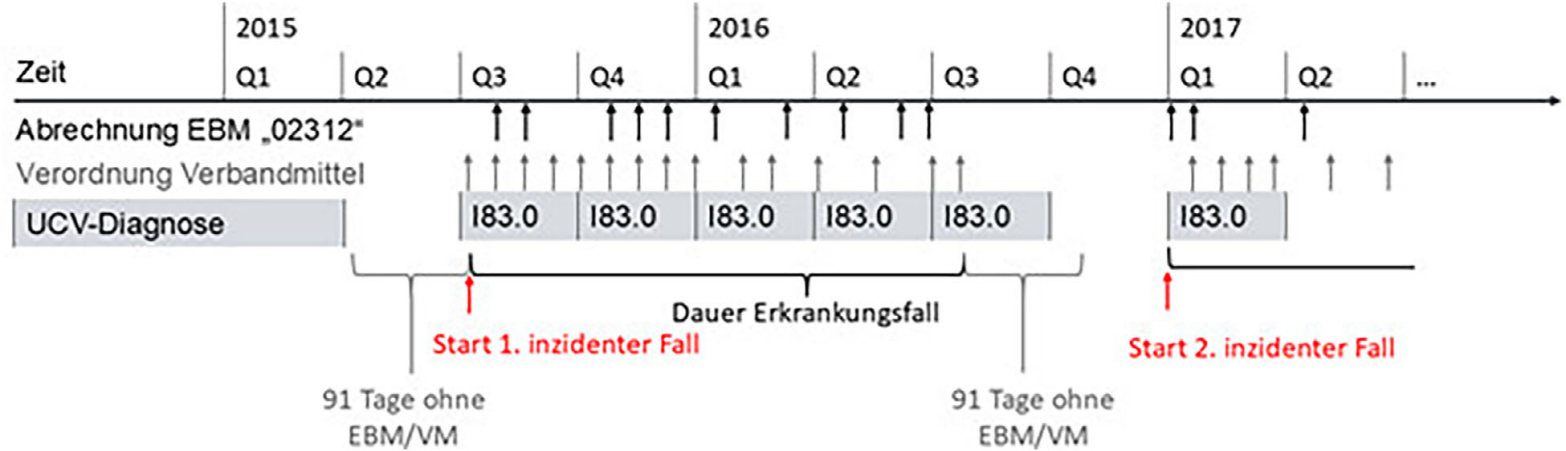
Bestimmung inzidenter und abgeschlossener Erkrankungsfälle anhand von Informationen zu Diagnosen, verordneten Verbandmitteln (VM) und der EBM‐Ziffer 02312 (EBM). Jeder Pfeil kennzeichnet das Datum einer Abrechnung der EBM‐Ziffer 02312 (obere Reihe) beziehungsweise einer Verbandmittelverordnung (untere Reihe). Die Diagnose eines Ulcus cruris venosum (in der Grafik beispielhaft dargestellt durch den ICD‐10‐Code I83.0) konnte ausschließlich quartalsbezogen und ohne exaktes Datum zugeordnet werden. Als Beginn eines inzidenten Krankheitsfalls wurde entweder das Datum der ersten Abrechnung der EBM‐Ziffer 02312 oder das Datum der ersten Verbandmittelverordnung definiert, sofern im selben Quartal eine UCV‐Diagnose dokumentiert war. Zur Abgrenzung einzelner Erkrankungsepisoden wurde ein Zeitraum von 91 Tagen zugrunde gelegt. Das Ende der Erkrankung (sofern bestimmbar) wurde definiert als das Datum der letzten Abrechnung der EBM‐Ziffer 02312 oder der letzten Verbandmittelverordnung, gefolgt von einem Zeitraum von mindestens 91 Tagen ohne weitere Verordnung oder Abrechnung.

### Zielkriterien

Als Zielkriterium wurde zunächst die Behandlung mit einer Kompressionstherapie untersucht. Kompressionsmittel, das heißt, medizinische Kompressionsstrümpfe, zweilagige Ulkus‐Strumpfsysteme, medizinisch adaptive Kompressionssysteme (MAK), intermittierende pneumatische Kompression (IPK) sowie Materialien für phlebologische Kompressionsverbände (zum Beispiel Mehrkomponentensysteme, Kurzzugbinden, Polsterbinden, Schlauchverbände) wurden anhand von Hilfsmittelnummern und einer PZN‐Liste mit insgesamt über 1100 Produkten identifiziert.

Um die Auswirkungen einer konsequenten Kompressionstherapie zu untersuchen, wurden Patienten mit konsequenter Kompressionstherapie mit Patienten, die eine unzureichende oder gar keine Kompressionstherapie erhielten, verglichen. Eine konsequente Kompressionstherapie auf Basis der Routinedaten wurde definiert als Verordnung eines Kompressionsmittels innerhalb von 21 Tagen nach Start des Erkrankungsfalls mit nachfolgender regelmäßiger Neuverordnung. Als regelmäßig galt eine Verordnung, wenn nach der Verordnung eines Kompressionsmittels innerhalb eines festgelegten Zeitraums entweder ein neues Kompressionsmittel verordnet wurde oder das Krankheitsende erreicht war. Für die Kompressionsbinden wurde diese Zeit auf 3 Monate festgelegt, für alle anderen Kompressionsmittel auf 7 Monate.

Als weitere Zielkriterien wurden die Häufigkeit von einschlägigen operativen Eingriffen, von erkrankungsspezifischen Hausarztkontakten und einschlägigen Facharztkontakten (Chirurgie, Gefäßchirurgie, Dermatologie oder Angiologie) untersucht.

Für die Ermittlung der Zielkriterien wurden jeweils die ersten 90 Tage der inzidenten Erkrankungsfälle analysiert. Um auch die gesamte Behandlungszeit zu untersuchen, wurden die Zielkriterien zudem für alle in der Beobachtungszeit abgeschlossenen Erkrankungsfälle ermittelt.

### Statistische Analyse

Soziodemografische Merkmale der Stichprobe sowie die Häufigkeit von Kompressionstherapie, operativen Eingriffen und ärztlichen Konsultationen (Haus‐ und Fachärzte) wurden deskriptiv ausgewertet. Die Wundheilungsdauer wurde für abgeschlossene Fälle berechnet.

Zur Untersuchung der Effekte einer konsequenten Kompressionstherapie wurden Fälle mit konsequenter Kompression mit Fällen ohne oder mit unzureichender Kompression verglichen. Für Gruppenvergleiche wurden zweiseitige t‐Tests für unabhängige Stichproben durchgeführt; Häufigkeiten wurden mittels χ^2^‐Test verglichen. Das Signifikanzniveau lag bei p = 0,05.

### Kostenanalyse

Für die gesundheitsökonomische Analyse wurden die Behandlungskosten aus Sicht der gesetzlichen Krankenversicherung und anhand der übermittelten fallbezogenen Abrechnungsdaten evaluiert. Die Kosten wurden für abgeschlossene Erkrankungsfälle innerhalb des Erkrankungszeitraums ermittelt und darüber hinaus auch für einen Zeitraum von 365 Tagen nach Erstdiagnose, um die Vergleichbarkeit mit vorangegangenen Studien zu gewährleisten. Dazu wurden für jeden Patienten die Kosten innerhalb des jeweiligen Zeitraums (Krankheitsdauer oder 365 Tage nach Erstdiagnose) summiert und die Durchschnittskosten als Summe der Kosten aller Patienten geteilt durch die Anzahl der Patienten berechnet. Anhand der vorliegenden fallbezogenen Abrechnungsdaten wurden die Kosten für ambulante ärztliche Versorgung, Hospitalisierung, die häusliche Krankenpflege sowie rezeptierte Medikamente und Hilfsmittel ermittelt. Die spezifischen Kosten für Verbands‐ und Kompressionsmittel wurden anhand der PZN und Hilfsmittelidentifikationsnummer identifiziert. Für krankheitsspezifische Ausgaben wurden die Kosten für ambulante Leistungen (Hausärzte, relevante Fachärzte in Dermatologie, Chirurgie, Gefäßchirurgie und Angiologie), Verbandmaterial, Kompressionsmaterial und ambulante Pflege kumuliert.

## ERGEBNISSE

### Studienpopulation

Die Ausgangspopulation für die Analysen umfasste fallbasierte Daten von 40 046 versicherten erwachsenen Personen, die in den Jahren 2015 bis 2019 mindestens einmal im ambulanten Bereich die Diagnose eines UCV erhielten. Deskriptive Statistiken zur Studienpopulation sind in Tabelle 1 zu finden. Es konnten 8092 inzidente Erkrankungsfälle in den Daten identifiziert werden, wovon 5782 Erkrankungsfälle zum Ende des Beobachtungszeitraums abgeschlossen waren.

**TABELLE 1:** Deskriptive Statistiken der Studienpopulation.

	Ausgangspopulation	Inzidente Erkrankungsfälle	Abgeschlossene Erkrankungsfälle
Anzahl Versicherte	40 046	8092	5782
Alter in Jahren (MW ± SD)	71,3 ± 14,5	76,6 ± 12,4	75,6 ± 12,5
Anteil weiblich/männlich	62,7 %/37,3 %	60,8 %/39,2%	60,8 %/39,2 %
Anteil mit Pflegegrad zu Krankheitsbeginn	–	40,9 %	37,2 %
Charlson‐Index^1^ zu Krankheitsbeginn (MW ± SD)	–	3,4 ± 2,7	3,1 ± 2,6
Anteil mit Kontraindikation für Kompressionstherapie^2^	–	6,31 %	5,38 %

*Abk*.: MW, Mittelwert; SD, Standardabweichung

^1^ Nach Glasheen et al., 2019^12^

^2^ Ambulante Diagnose einer dekompensierten Herzinsuffizienz (I50.14, I50.05) oder einer peripheren arteriellen Verschlusskrankheit in Stadium IV (I70.24, I70.25)

### Versorgung von Patienten mit UCV

Von den erwachsenen Versicherten mit Neuerkrankung und ohne Kontraindikation für eine Kompressionstherapie (ambulante Diagnose einer dekompensierten Herzinsuffizienz [I50.14, I50.05] oder peripherer arterieller Verschlusskrankheit im Stadium IV [I70.24, I70.25], n = 7581), erhielten 47,3 % (n = 3589) innerhalb der ersten 90 Tage nach Krankheitsbeginn mindestens ein Kompressionshilfsmittel. Die erste Verordnung erfolgte im Durchschnitt nach 24,3 ± 27,0 Tagen. Bei 27,5 % (n = 2084) der Versicherten wurde das erste Kompressionsprodukt innerhalb der ersten 21 Tage verschrieben. Abbildung 2 zeigt die Fallzahlen zur Versorgung mit Kompressionstherapie.

**ABBILDUNG 2:**
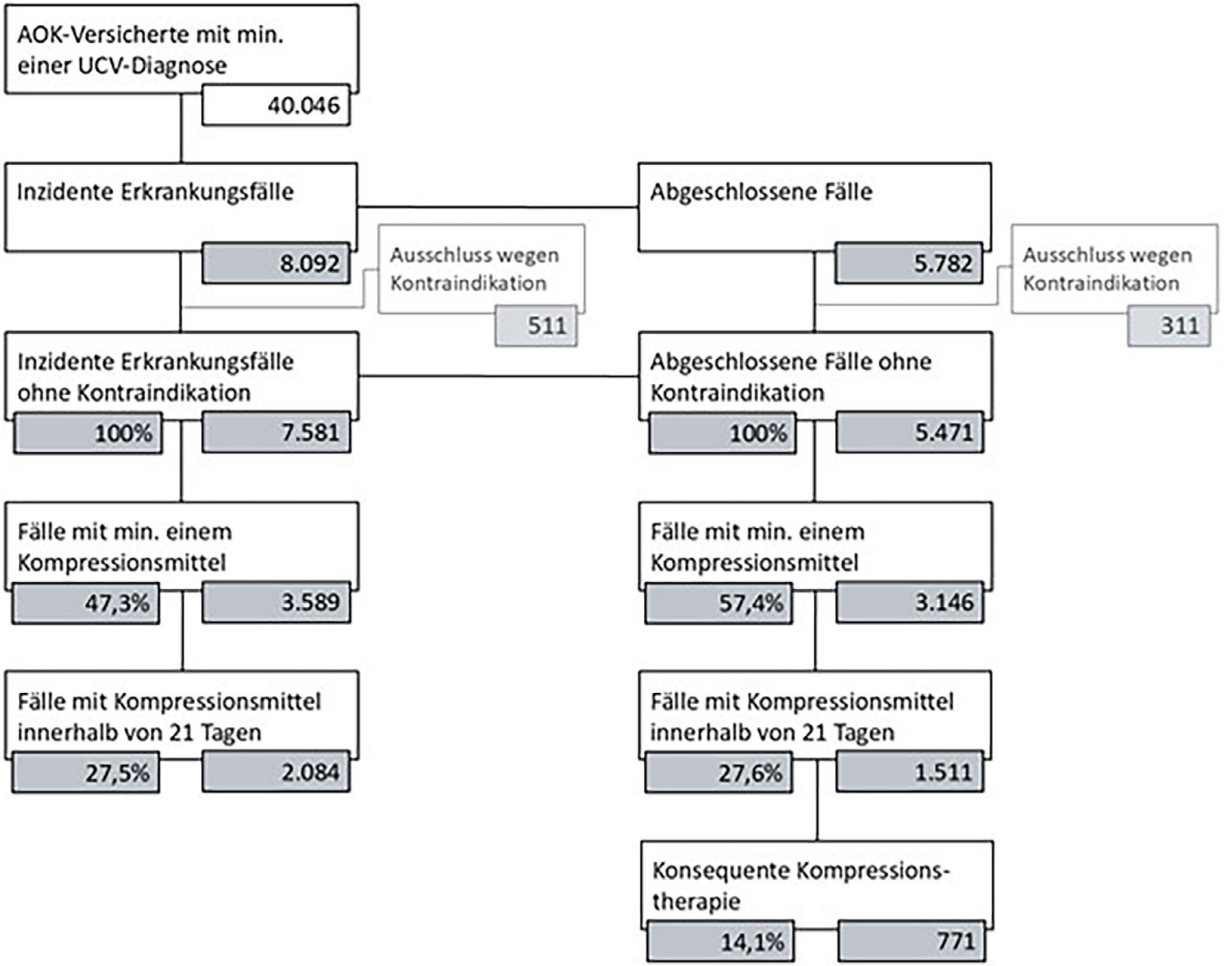
Stichprobengrößen und Anteile (in %) der Patienten mit Versorgung durch Kompressionstherapie.

Patienten ohne Kontraindikation, bei denen das Auftreten und die Abheilung eines UCV vollständig innerhalb des Beobachtungszeitraums lag (n = 5471), erhielten in 57,4 % (n = 3146) der Fälle während der Krankheitsdauer mindestens ein Kompressionsprodukt. In 27,6 % (n = 1511) der Fälle wurde ein Kompressionshilfsmittel innerhalb der ersten 21 Tage verordnet. Bei 14,1 % (n = 771) wurde eine konsequente Kompressionstherapie im Sinne der Studiendefinition über den gesamten Krankheitszeitraum durchgeführt.

Darüber hinaus wurden bei n = 135 Versicherten mit abgeschlossener Erkrankung (2,3 %) invasive Verfahren zur Ausschaltung von Krampfadern durchgeführt. Bei n = 126 dieser 135 Personen (93,3 %) erfolgte eine Ablation der Stammvenen, und 21,5 % (n = 29) unterzogen sich einer endovenösen Ablation. Insgesamt wurden bei n = 94 der Versicherten mit Neuerkrankung (1,2 %) die Eingriffe innerhalb der ersten 90 Tage nach Krankheitsbeginn durchgeführt.

Die Mehrheit der Patienten mit Neuerkrankung wurde überwiegend von Hausärzten behandelt (74,2 %). Über die Hälfte (60,9 %) wurde ausschließlich hausärztlich betreut, 16,6 % überwiegend fachärztlich. Innerhalb von 90 Tagen nach Erstdiagnose suchten Patienten durchschnittlich sechsmal einen Arzt auf. Personen mit konsequenter Kompressionstherapie wurden häufiger überwiegend von Fachärzten betreut (23,3 %) und seltener überwiegend hausärztlich (64,7 %) als Patienten ohne konsequente Kompressionstherapie (überwiegend Fachärzte: 16,1 %, überwiegend Hausärzte: 76,3 %; jeweils p < 0,001).

### Wundheilung

Die durchschnittliche Wundheilungsdauer bei abgeschlossenen Fällen betrug 178,9 ± 157,8 Tage (Median: 125 Tage). Die Heilungsrate innerhalb von 6 Monaten nach Krankheitsbeginn lag bei 69,4 %, innerhalb von 12 Monaten bei 90,6 %. Versicherte, die eine konsequente Kompressionstherapie erhielten, wiesen eine kürzere Wundheilungsdauer auf (121,6 ± 86,2 Tage) im Vergleich zum restlichen Kollektiv (188,1 ± 164,2 Tage), p < 0,001. Auch die Heilungsraten nach 6 und 12 Monaten waren bei Versicherten mit konsequenter, regelmäßiger Kompressionstherapie höher (6 Monate: 85,9 %, 12 Monate: 97,1 %) als in der übrigen Gruppe (6 Monate: 66,7 %, 12 Monate: 85,9 %), p < 0,001.

### Kostenanalyse

Während der Krankheitsdauer beliefen sich die durchschnittlichen Gesamtkosten pro Patient auf 5622 ± 9255 €, die krankheitsspezifischen Kosten auf 2779 ± 4862 €. Die durchschnittlichen jahresbezogenen Gesamtkosten pro Patient lagen bei 8294 ± 10 805 €, die krankheitsspezifischen Kosten bei 3840 ± 5566 €.

Der Kostenvergleich zwischen Patienten mit und ohne konsequente Kompressionstherapie zeigte während der Krankheitsdauer niedrigere Gesamtkosten (p < 0,001) und niedrigere krankheitsspezifische Kosten (p = 0,017) für die Gruppe mit konsequenter Kompressionstherapie (4280 ± 5326 € bzw. 2498 ± 2785 €) (Tabelle 2) im Vergleich zu Patienten ohne oder mit unzureichender Kompressionstherapie (5683 ± 9657 € beziehungsweise 2797 ± 5145 €, bei insgesamt kürzerer Behandlungsdauer in der Gruppe mit konsequenter Therapie. Wurden die Kosten bezogen auf ein Jahr ab Erstdiagnose verglichen, waren die Gesamtkosten beider Gruppen ähnlich (7933 ± 10 247 € beziehungsweise 8019 ± 10 507 €, p = 0,84, während die krankheitsspezifischen Kosten bei Patienten mit konsequenter Kompressionstherapie leicht höher lagen als bei jenen ohne oder mit unzureichender Therapie (4292 ± 4070 € beziehungsweise 3682 ± 5674 €, p = 0,006. Eine detaillierte Aufschlüsselung der Kosten nach einzelnen Bereichen des Gesundheitssystems ist in Tabelle 2 (Kosten während der Krankheitsdauer) und Tabelle 3 (Jahreskosten ab Krankheitsbeginn) dargestellt.

**TABELLE 2:** Kosten der Behandlung eines Versicherten mit Ulcus cruris venosum (UCV) innerhalb der Erkrankungsdauer in Euro (Median [IQR^0^]).

	Gesamt	Personen ohne Kontraindikation für Kompressionstherapie	
Kostenbereich:	n = 5782	*Kompressionstherapie* ^a^ *n = 771*	*Keine/unzureichende Kompressionstherapie* ^b^ *n = 4700*
Ambulant	450 (254–826)	364 (209–617)	460 (263–853)
Davon Hausarzt	242 (130–428)	175 (100–312)	252 (136–451)
Davon Facharzt^1^	0 (0–89)	0 (0–88)	0 (0–89)
Ambulante Pflege	0 (0–980)	0 (0–1150)	0 (0–920)
Arzneimittelverordnungen	1087 (539–2160)	1016 (558–1899)	1 082 (522–2197)
Davon Verbandmittel	436 (198–968)	444 (210–869)	434 (193–984)
Davon Kompressionsmittel^2^	45 (0–139)	178 (90–408)	19 (0–104)
Krankenhaus	0 (0–1297)	0 (0–0)	0 (0–1240)
Hilfsmittel	76 (0–239)	87 (0–225)	67 (0–234)
Erkrankungsspezifische Kosten	1502 (730–3187)	1689 (901–2999)	1 446 (696–3214)
Gesamtkosten	2888 (1271–6559)	2583 (1330–5139)	2864 (1220–6603)

^0^ IQR: Interquartilsabstand

^1^ Dermatologie, Chirurgie, Gefäßchirurgie und Angiologie

^2^ Kosten für Kompressionsmittel sind teilweise in den Arzneimittelverordnungen und teilweise in den Hilfsmitteln verortet.

^a^ Patienten mit konsequenter Kompressionstherapie

^b^ Patienten ohne Kompressionsmittel oder mit unzureichender Kompressionstherapie

**TABELLE 3:** Kosten der Behandlung eines Versicherten mit Ulcus cruris venosum (UCV) im Zeitraum von 365 Tagen nach Krankheitsbeginn in Euro (Median [IQR^0^]).

	Gesamt	Personen ohne Kontraindikation für Kompressionstherapie	
Kostenbereich:	n = 5451	*Kompressionstherapie* ^a^ *n = 719*	*Keine/unzureichende Kompressionstherapie* ^b^ *n = 4448*
Ambulant	1 002 (667–1495)	964 (665–1473	1000 (658–1485)
Davon Hausarzt	532 (388–762)	505 (374–732)	532 (386–762)
Davon Facharzt^1^	24 (0–129)	33 (0–138)	20 (0–127)
Ambulante Pflege	0 (0–1496)	0 (0–2440)	0 (0–1316)
Arzneimittelverordnungen	1958m (1056–3275)	1954 (1043–3286)	1921 (1034–3210)
Davon Verbandmittel	514 (239–1050)	506 (256–999)	509 (230–1062)
Davon Kompressionsmittel^2^	52 (0–186)	264 (132–610)	0 (0–139)
Krankenhaus	0 (0–1546)	0 (0–0)	0 (0–1426)
Hilfsmittel	105 (0–486)	159 (0–545)	103 (0–466)
Erkrankungsspezifische Kosten	2413 (1429–4915)	3032 (1696–5674)	2308 (1379–4705)
Gesamtkosten	5202 (2735–10 243)	5264 (2861–9528)	5044 (2634–10 033)

^0^ IQR: Interquartilsabstand

^1^ Dermatologie, Chirurgie, Gefäßchirurgie und Angiologie

^2^ Kosten für Kompressionsmittel sind teilweise in den Arzneimittelverordnungen und teilweise in den Hilfsmitteln verortet.

^a^ Patienten mit konsequenter Kompressionstherapie

^b^ Patienten ohne Kompressionsmittel oder mit unzureichender Kompressionstherapie

## DISKUSSION

### Versorgung von Patienten mit UCV

Ziel dieser Studie war es, die aktuelle Versorgungssituation erwachsener Patienten mit UCV darzustellen – insbesondere im Hinblick auf den Einsatz kausal orientierter Behandlungsansätze wie die Umsetzung einer phlebologischen Kompressionstherapie oder chirurgischer Verfahren zur Behandlung der zugrunde liegenden venösen Hypertonie. Die Ergebnisse legen nahe, dass die phlebologische Kompressionstherapie in der Versorgung von UCV‐Patienten selbst bei fehlenden Kontraindikationen in vielen Fällen gar nicht oder nicht konsequent angewendet wird. Weniger als die Hälfte der Patienten erhielt innerhalb der ersten 90 Tage nach Krankheitsbeginn ein Kompressionshilfsmittel. Weniger als jede fünfte Person erhielt eine konsequente, regelmäßige und frühzeitige Kompressionstherapie. Eine invasive Ablation der Stammvenen wurde nur bei etwa 2 % der Patienten durchgeführt. Insgesamt belegen die Daten somit deutlich, dass Patienten mit UCV nicht adäquat behandelt wurden.

Hinsichtlich des Einsatzes von Kompressionshilfsmitteln bei Patienten mit UCV stimmen die Ergebnisse mit früheren Versorgungsanalysen überein, die Anwendungsraten von etwa 40 % zeigten.^8^, ^9^ Darüber hinaus deutet die in dieser Analyse vorgenommene Modellierung des Krankheitsverlaufs jedoch darauf hin, dass – entgegen den aktuellen Leitlinienempfehlungen – eine Kompressionstherapie nur in sehr wenigen Fällen frühzeitig und konsequent eingesetzt wird.^1^ Auch kausal orientierte invasive Verfahren wie die Ablation von Stammvenen werden in Deutschland selten durchgeführt. Auf Grundlage der analysierten Daten ist daher davon auszugehen, dass mehr als 80 % der Patienten keine ausreichende ursächlich orientierte Behandlung erhalten. Daraus ergibt sich ein dringender Handlungsbedarf zur Umsetzung der aktuellen Leitlinienempfehlungen in der Versorgung von Menschen mit UCV. Studien zeigen, dass eine konsequente Kompressionstherapie zu einer Verkürzung der Wundheilungsdauer und einer Reduktion der Rezidivrate führt.^6^, ^7^, ^13^ Dies wird auch durch die vorliegende Auswertung bestätigt: Patienten mit konsequenter, regelmäßiger und frühzeitiger Kompressionstherapie wiesen eine kürzere Wunddauer auf als Patienten ohne eine solche Therapie.

Die Gründe für eine inkonsequente oder vollständig fehlende Kompressionstherapie können vielfältig sein. Ein möglicher Grund für die niedrige Anwendungsrate ist ein mangelndes Wissen über die Relevanz der Kompressionstherapie bei Leistungserbringenden im Gesundheitswesen.^13^, ^14^ Auch eine unzureichende Aufklärung der Patienten trägt dazu bei, dass die Kompressionstherapie nicht begonnen oder nicht fortgeführt wird.^15^, ^16^ Darüber hinaus kann die Beteiligung vieler verschiedener Akteuren in der Versorgung dazu führen, dass keine konsequente Kompressionstherapie erfolgt und Patienten eine eher passive Rolle einnehmen.^14^

Die Gründe für eine inkonsequente oder vollständig fehlende Anwendung der Kompressionstherapie können vielfältig sein. Ein möglicher Grund für die niedrige Anwendungsrate ist ein mangelndes Wissen über die Relevanz der Kompressionstherapie bei Leistungserbringenden im Gesundheitswesen.^13^, ^14^ Auch eine unzureichende Aufklärung der Patienten trägt dazu bei, dass die Kompressionstherapie nicht begonnen oder nicht fortgeführt wird.^15^, ^16^ Darüber hinaus kann die Beteiligung vieler verschiedener Akteuren in der Versorgung dazu führen, dass keine konsequente Kompressionstherapie erfolgt und Patienten eine eher passive Rolle einnehmen.^14^ Patientenzentrierte Ansätze, insbesondere die Schulung von Patienten mit UCV, wirken sich positiv auf die Therapieadhärenz und die Wundheilung aus und sollten daher als integraler Bestandteil der ambulanten Versorgung chronischer Wunden betrachtet werden.^17^, ^18^ Die Ergebnisse dieser Studie bestätigen auch, dass die überwiegende Mehrheit der Patienten mit UCV hausärztlich angebunden ist. Die Umsetzung möglicher Lösungsansätze – etwa durch Kompetenzsteigerung oder die Förderung patientenzentrierter Behandlungsansätze – erscheint daher besonders im Bereich der hausärztlichen Versorgung sinnvoll. Die Kompetenz zur Indikationsprüfung und Durchführung der Kompressionstherapie bei allen in die Versorgung chronischer Wunden eingebundenen Akteuren gefördert werden. Insbesondere besteht auch Optimierungsbedarf bei der korrekten praktischen pflegerischen Umsetzung, zum Beispiel durch ambulante Pflegedienste.^19^ Um die Anwendung einer leitliniengerechten Kompressionstherapie gezielt zu fördern, sind zudem weitere Studien erforderlich, die sich mit den bestehenden Barrieren befassen.

### Kostenanalyse

In der vorliegenden Studie wurden die Kosten der Erkrankung aus Sicht der gesetzlichen Krankenversicherung berechnet. Die durchschnittlichen patientenbezogenen Gesamtkosten beliefen sich auf rund 5600 € während der Krankheitsdauer beziehungsweise auf rund 8300 € pro Jahr. Diese Werte liegen somit über den Ergebnissen früherer Studien, die von jährlichen Gesamtkosten in Höhe von etwa 4000 € ausgingen.^20^, ^21^ Der Vorteil der hier gewählten Kostenberechnung auf Grundlage der konkreten individuellen Krankheitsdauer im Vergleich zu früheren Studien besteht darin, dass die krankheitsspezifischen Kosten des UCV besser abgeschätzt werden können. Allerdings hat dieser Ansatz auch den Nachteil, dass stationäre Kosten nicht adäquat berücksichtigt werden können, da Patienten mit langen Krankenhausaufenthalten aus methodischen Gründen ausgeschlossen werden mussten, um die Krankheitsdauer zu bestimmen. Patienten mit konsequenter, regelmäßiger und frühzeitiger Kompressionstherapie wiesen während der Krankheitsdauer niedrigere Gesamtkosten und geringere krankheitsspezifische Kosten auf als andere Patienten. In der Zwölfmonatsanalyse hielt die anfängliche Kostenersparnis jedoch nicht an, vermutlich aufgrund der Überlagerung durch die ambulanten Gesamtkosten und bestehende Komorbiditäten.

Dennoch deuten die Ergebnisse darauf hin, dass durch eine schnellere Wundheilung im Rahmen einer Kompressionstherapie Kosteneinsparungen erzielt werden können – was plausibel erscheint. Dies gilt auch für indirekte Kosten, etwa durch krankheitsbedingte Arbeitsunfähigkeit, die in dieser Analyse nicht berücksichtigt wurden. Darüber hinaus ist davon auszugehen, dass auch die Reduktion des Bedarfs an Verbandmaterialien, die mit knapp 1000 € einen hohen Anteil an den krankheitsspezifischen Kosten ausmachen, erheblich zur Kostensenkung beiträgt. Dieser Aspekt gewinnt besonders an Bedeutung, da aufgrund des demografischen Wandels in den nächsten 10–20 Jahren mit einem deutlichen Anstieg der Inzidenz chronischer Wunden zu rechnen ist.^22^

### Limitationen

Im Hinblick auf die Ergebnisse der vorliegenden Studie ist zu beachten, dass die zugrunde liegenden analysierten Daten zu Abrechnungszwecken erhoben wurden. Die Abrechnungsdaten zum UCV enthalten keine spezifischen krankheitsbezogenen Parameter wie zum Beispiel die CEAP‐Klassifikation oder Wundgröße. Daher kann nicht unterschieden werden, ob Patienten mit großen oder komplizierten Wunden möglicherweise häufiger eine Kompressionstherapie erhalten haben. Ein solcher Bias könnte auch erklären, warum sich die Kostendifferenzen zwischen Fällen mit konsequenter Kompressionstherapie und dem übrigen Kollektiv nach einem Jahr nicht mehr nachweisen lassen. Ein Erfassungsdefizit ist eine weitere mögliche Limitation von Abrechnungsdaten. Dies betrifft sowohl die Identifikation von UCV‐Fällen über ICD‐10‐Codes, wie in dieser Studie erfolgt, als auch die Ermittlung der krankheitsspezifischen Kosten. Nicht alle Kosten können eindeutig der Erkrankung zugeordnet werden, da spezifische Abrechnungsziffern häufig fehlen oder – wie zum Beispiel der EBM‐Code 02312 – zu selten verwendet werden. Solche Verzerrungen dürften jedoch für beide Gruppen – mit und ohne konsequente Kompressionstherapie – in ähnlicher Weise auftreten und ermöglichen daher dennoch einen vergleichbaren Gruppenvergleich. Die Kosten für Verbände und Kompressionsprodukte lassen sich relativ eindeutig der Erkrankung zuordnen und dürften nur in Einzelfällen fehlerhaft zugewiesen worden sein. In Bezug auf invasive Verfahren zur Behandlung des oberflächlichen Venenrefluxes ist zu berücksichtigen, dass endovenöse Ablationen in Deutschland nicht regelmäßig von der gesetzlichen Krankenversicherung erstattet werden.

### Schlussfolgerungen

Die vorliegende Analyse zeigt, dass in der ambulanten Versorgung des UCV rund 80 % der Patienten weder eine konsequente Kompressionstherapie noch eine operative Behandlung erhalten und somit nicht adäquat versorgt sind. Es besteht somit ein dringender Handlungsbedarf zur Verbesserung der Behandlung von Patienten mit UCV in Deutschland. Lösungsansätze sollten darauf abzielen, die Kompetenzen der beteiligten Akteure zu stärken und patientenzentrierte Behandlungsansätze – insbesondere in der primärärztlichen und ambulanten Versorgung – zu fördern. Es besteht ein klarer Bedarf an einem Bewusstseinswandel: weg von einer rein lokal orientierten Wundversorgung hin zu einer umfassenden, kausal ausgerichteten Therapie des UCV. Die konsequente Umsetzung ursachenbezogener Behandlungsstrategien könnte eine langfristige Chronifizierung der Wundsituation verhindern und die Lebensqualität vieler betroffener Patienten deutlich verbessern.

## DANKSAGUNG

Open access Veröffentlichung ermöglicht und organisiert durch Projekt DEAL.

## FÖRDERUNG

Das Projekt *Ulcus Cruris Care* wird durch den Innovationsausschuss des Gemeinsamen Bundesausschusses (G‐BA) gefördert (01VSF19043).

## INTERESSENKONFLIKT

K.P. erhielt Honorare für Beratungen und/oder Vorträge von Bauerfeind, Essity/Jobst, Curea, Hartmann, Lohmann & Rauscher, Juzo, medi und Urgo. Alle weiteren Autoren erklären, dass kein Interessenkonflikt besteht.
